# Supplementary material for: Comparative evaluation of Olink Explore 3072 and mass spectrometry with peptide fractionation for plasma proteomics
Source: Commun Chem. 2025 Nov 4;8:327. doi: 10.1038/s42004-025-01753-2 (PMC12586489; doi:10.1038/s42004-025-01753-2)
Supplement: Supplementary file 2 — Supplementary Information [file 42004_2025_1753_MOESM2_ESM.pdf]

Supplementary Materials for

Comparative evaluation of Olink Explore 3072 and mass spectrometry with peptide fractionation for plasma proteomics

Authors

Noora Sissala[1], Haris Babačić[1], Isabelle R. Leo[1,2], Xiaofang Cao[1], Jenny Forshed[1,3], Lars E. Eriksson[4,5], Janne Lehtiö[1], Claudia Fredolini[6,7], Mikael Åberg[8,9], Maria Pernemalm[1]

Affiliations

1. Department of Oncology-Pathology, Karolinska Institutet and Science for Life Laboratory, Solna, Sweden
2. Department of Immunobiology, Yale School of Medicine, New Haven, CT, USA
3. Capio Elderly and Mobile Care, Stockholm, Sweden
4. Department of Neurobiology, Care Sciences and Society, Karolinska Institutet, Huddinge, Sweden
5. School of Health and Medical Sciences, City St George's, University of London, London, United Kingdom
6. Department of Protein Science, School of Engineering Sciences in Chemistry, Biotechnology and Health, KTH Royal Institute of Technology, Stockholm, Sweden
7. Affinity Proteomics Unit, Science for Life Laboratory, Solna, Sweden
8. Department of Medical Sciences, Uppsala University, Uppsala, Sweden
9. Affinity Proteomics Unit, Science for Life Laboratory, Uppsala, Sweden

Corresponding authors: Noora Sissala (noora.sissala@ki.se) and Maria Pernemalm (maria.pernemalm@ki.se)

This PDF file includes:

- Figures S1 to S19
- Tables S1 and S2

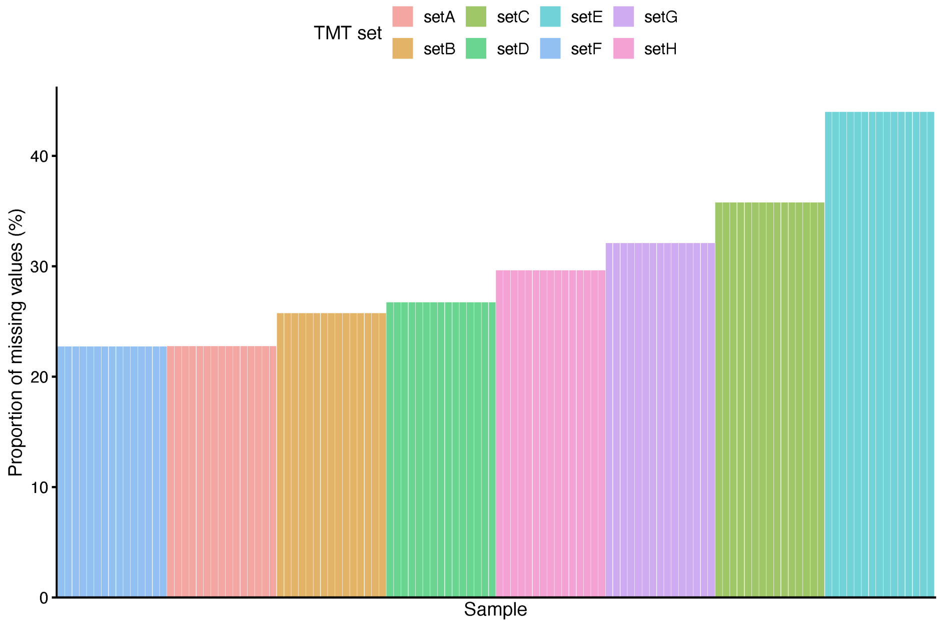

Figure S1. Proportion of missing values per sample in the MS data. The y-axis shows the proportion of missing values per sample (N = 120) in the HiRIEF LC-MS/MS data, with bars colored by tandem mass tag (TMT) set.

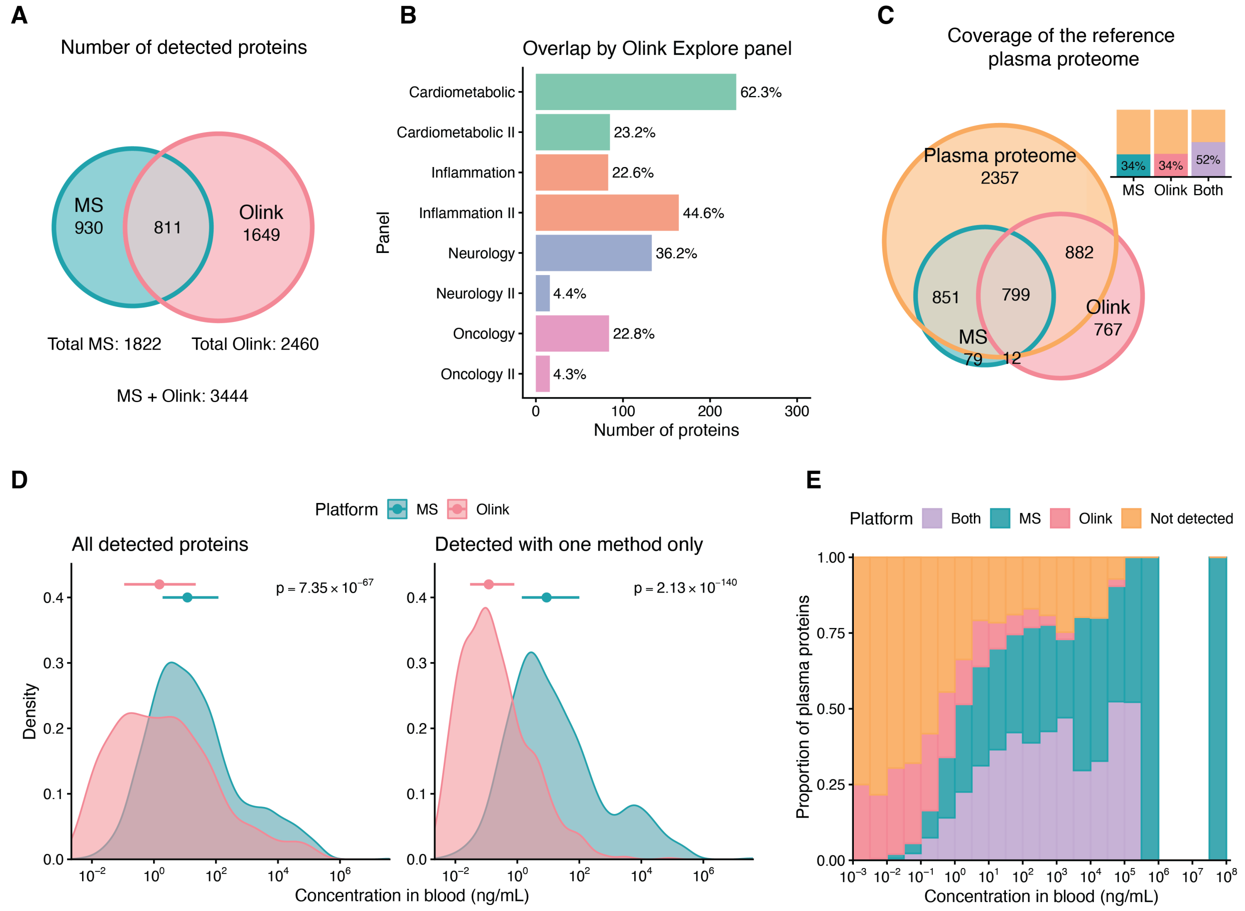

Figure S2. Detected proteins and proteome coverage after excluding proteins with >50% missing values. (A) Venn diagram of proteins detected in ≥50% of samples with HiRIEF LC-MS/MS and/or Olink Explore 3072, based on unique UniProt IDs. **(B)** Number and percentage of Olink assays in each Olink Explore panel detected with both Olink and MS. **(C)** Venn diagram comparing proteins detected by MS and Olink to proteins in the reference human plasma proteome, compiled from the Human Plasma Proteome Project (HPPP) database and the Human Protein Atlas (HPA) (see Methods). **(D)** Distribution of the estimated concentrations, from the HPA, of all detected proteins and proteins detected exclusively by MS or Olink. Medians and interquartile ranges (IQR) are indicated with points and error bars. Differences in protein concentration between platforms were tested using a two-sided Wilcoxon rank-sum test. **(E)** Plasma proteome coverage by estimated protein concentration. Each bar shows the proportion of proteins in the reference plasma proteome, within a specific concentration interval, that were detected with either MS only, Olink only, both methods, or neither method ("Not detected"). The x-axis intervals are right-closed.

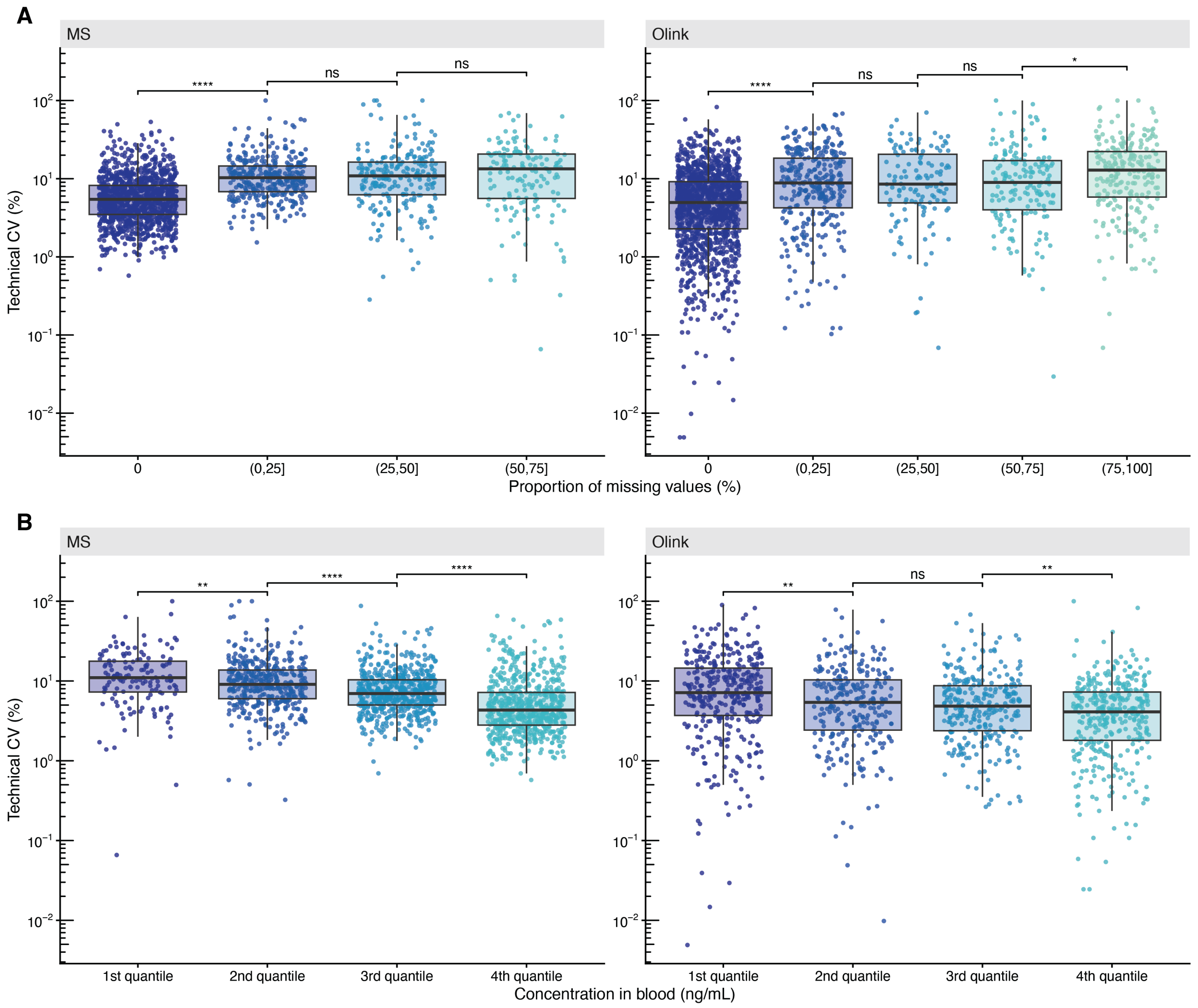

Figure S3. Relationship between technical CVs, missing values, and protein concentration. (A) Boxplots showing the distribution of technical coefficients of variation (CVs) by the proportion of missing values per protein. **(B)** Boxplots showing the distribution of technical CVs versus estimated blood concentration from the HPA. Concentrations were divided into quantiles based on the estimated concentrations of all detected proteins. The x-axis intervals therefore correspond to [0.002, 0.2], (0.2, 2], (2, 27] and (27, 4.0×10^7] ng/mL. In both panels, the y-axes are presented on a $\log_{10}$-scale, and p-values were determined using a two-sided Wilcoxon rank-sum test. P-values were adjusted using the false discovery rate (FDR) method. ns = not significant, * = $p < 0.05$, ** = $p < 0.01$, *** = $p < 0.001$, **** = $p < 0.0001$.

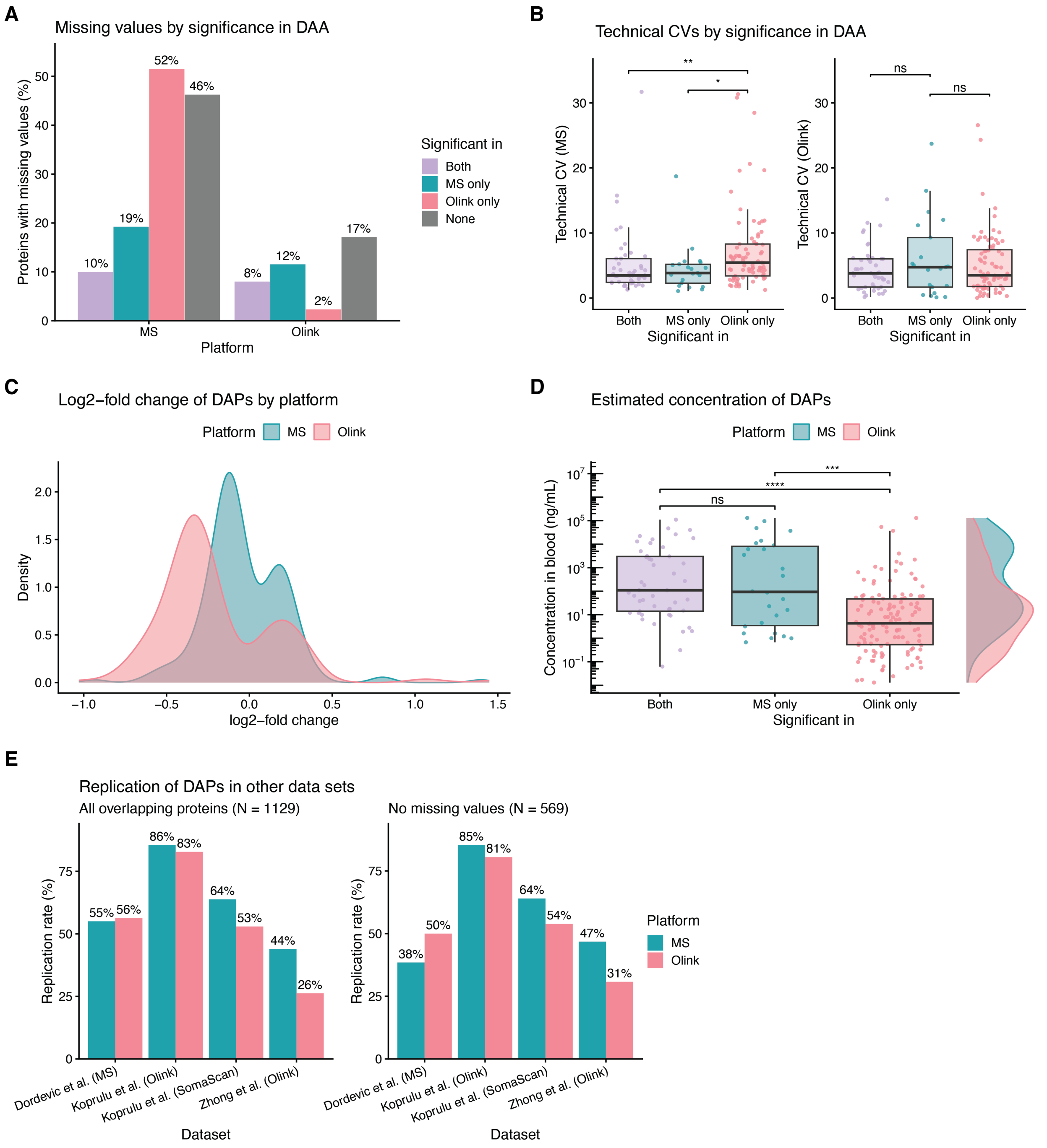

Figure S4. Additional differential abundance analysis (DAA) results. (A) Proportion of proteins with missing values in HiRIEF LC-MS/MS and Olink Explore 3072 data by statistical significance in DAA comparing plasma protein levels of all overlapping proteins between males and females (N = 1129, two-sided Welch's t-test, FDR < 0.05). "Olink only" = differentially abundant with Olink but not MS. "MS only" = differentially abundant with MS but not Olink. "Both" = differentially abundant in both datasets. **(B)** Boxplots showing the distribution of technical CVs for MS and Olink proteins relative to statistical significance in the DAA involving all overlapping proteins. P-values were determined using a two-sided Wilcoxon rank-sum test. ns = not

significant, * = $p < 0.05$, ** = $p < 0.01$. **(C)** Distribution of log2-fold change values between males and females (calculated as female-male) for differentially abundant proteins (DAPs) identified in each dataset. **(D)** Estimated blood concentration, from the HPA, for DAPs found by MS only, Olink only, or both platforms. P-values were determined using a two-sided Wilcoxon rank-sum test and adjusted for multiple testing with the FDR method. ns = not significant, *** = $p < 0.001$, **** = $p < 0.0001$. The density plot on the right shows the distribution of estimated protein concentration by platform for all DAPs. **(E)** Replication of MS and Olink DAPs from the present study in previous datasets reporting plasma proteome differences between males and females. The replication rate was calculated as the proportion of MS or Olink DAPs that were statistically significant with the same direction of difference in both datasets.

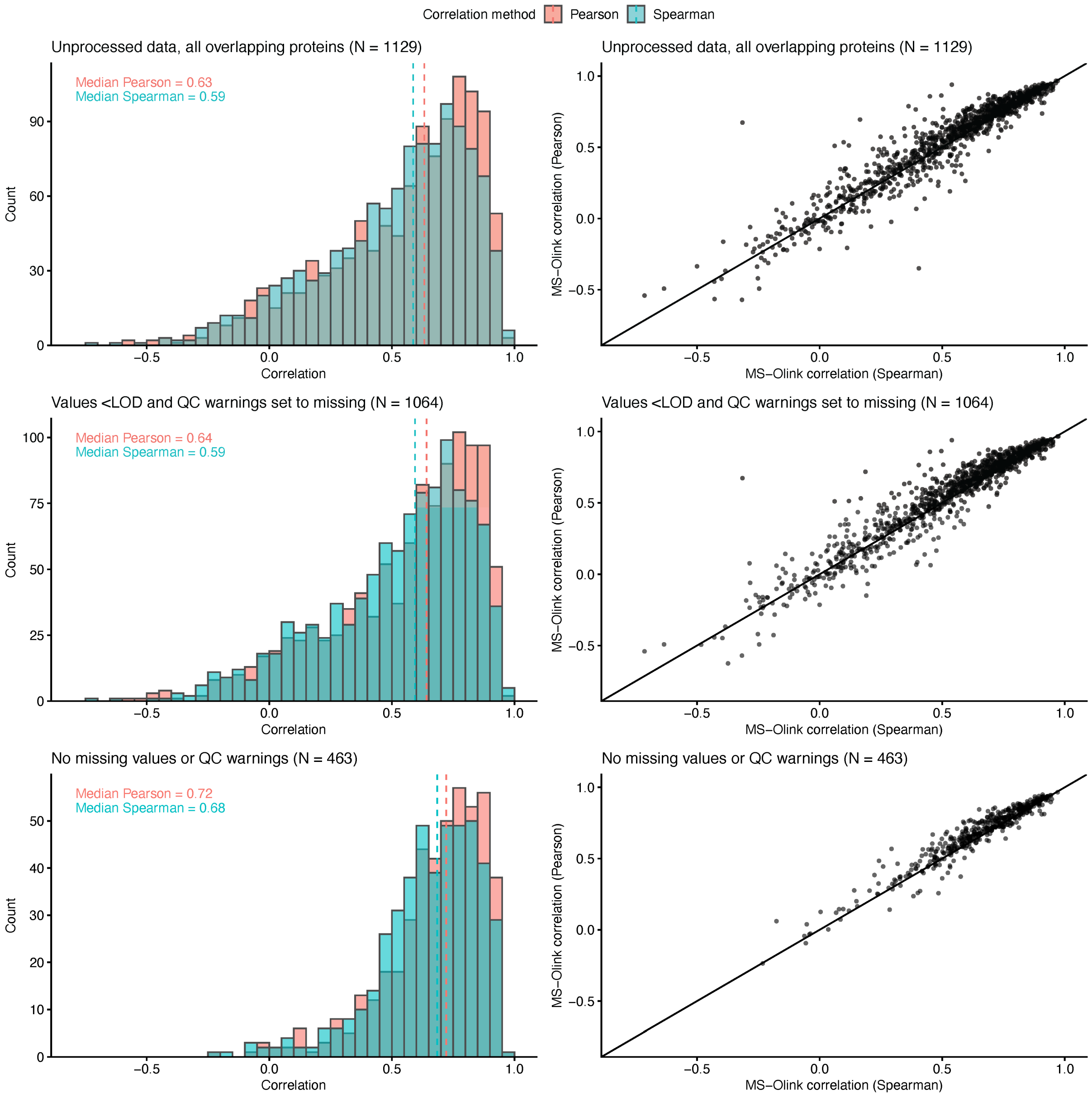

Figure S5. Comparison of Pearson and Spearman correlation coefficients. Histograms and scatter plots showing per-protein Pearson and Spearman correlation coefficients between HiRIEF LC-MS/MS and Olink Explore 3072 measurements for matching proteins. Results are shown for the unprocessed dataset including all overlapping proteins (N = 1129), a cleaned dataset where values below the limit of detection (LOD) and quality control (QC) warnings were set to missing (N = 1064), and a dataset of proteins with no missing values, values <LOD, or QC warnings (N = 463). A minimum of eight overlapping data points were required to calculate correlations.

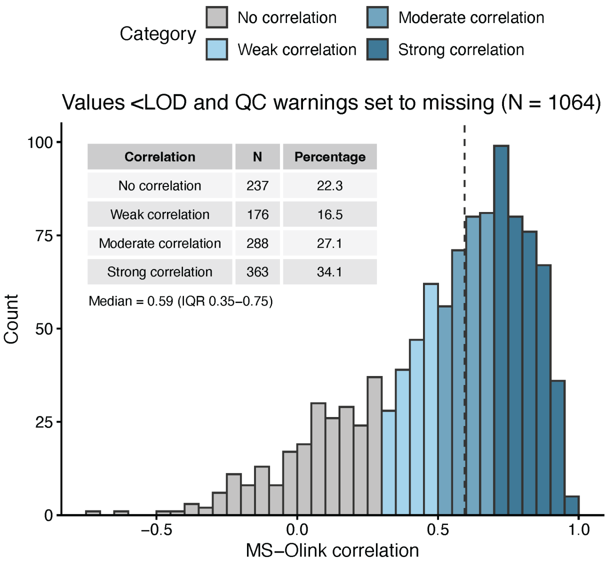

Figure S6. Cross-platform correlation of protein levels on filtered data. Histograms of per-protein Spearman correlations between HiRIEF LC-MS/MS and Olink Explore 3072 measurements for matching proteins, shown for a cleaned dataset where values <LOD and QC warnings in the Olink data were set to missing (N = 1064). A minimum of eight overlapping data points were required to calculate correlations. Correlations were categorized as no correlation: $\rho \in [-1, 0.3)$; weak correlation: $\rho \in [0.3, 0.5)$; moderate correlation: $\rho \in [0.5, 0.7)$; and strong correlation: $\rho \in [0.7, 1.0]$. The dashed line indicates the median correlation.

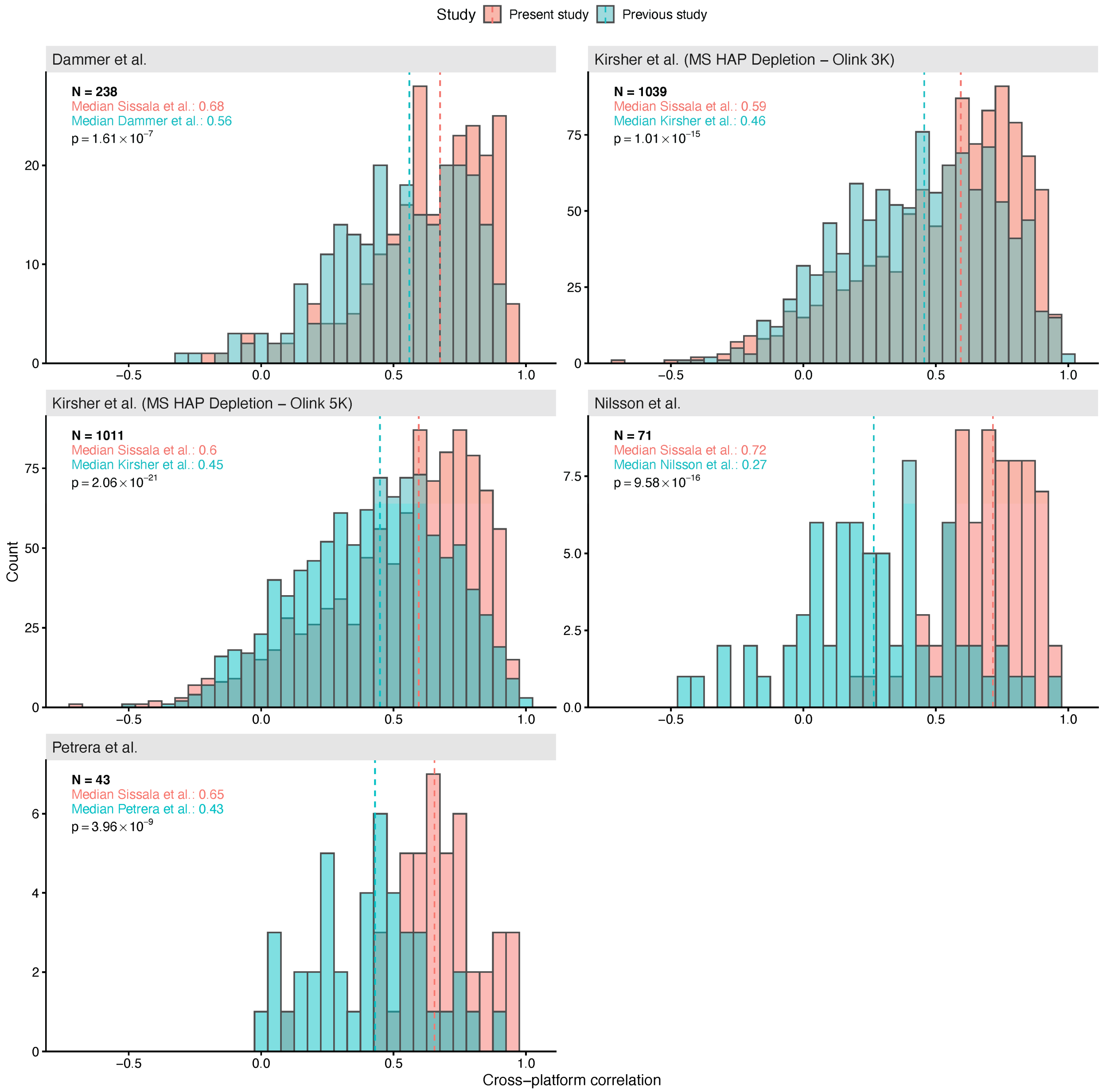

Figure S7. Comparison of MS-Olink correlations across studies. Histograms showing per-protein MS-Olink correlations from the present study and previous studies for matching protein pairs. The number of matching protein pairs and median cross-platform correlations for each study are indicated on each plot. Dashed lines represent the medians. P-values were determined using a two-sided Wilcoxon rank-sum test and adjusted with the FDR method. More details on the studies are given in Supplementary Data 10, and source data are provided in Supplementary Data 11. HAP Depletion = high-abundant protein depletion. Olink 3K = Olink Explore 3072. Olink 5K = Olink Explore HT.

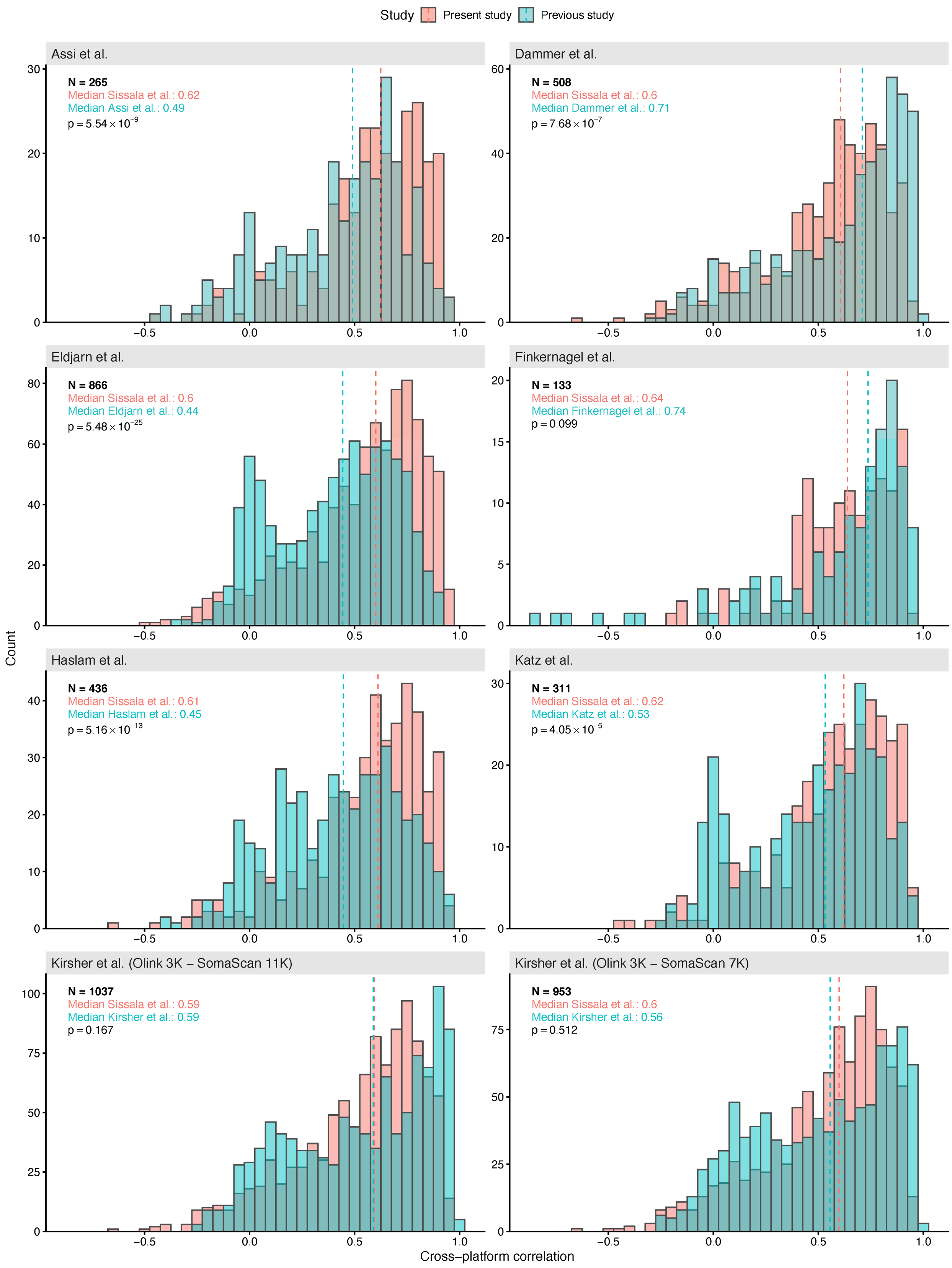

Continued on the next page

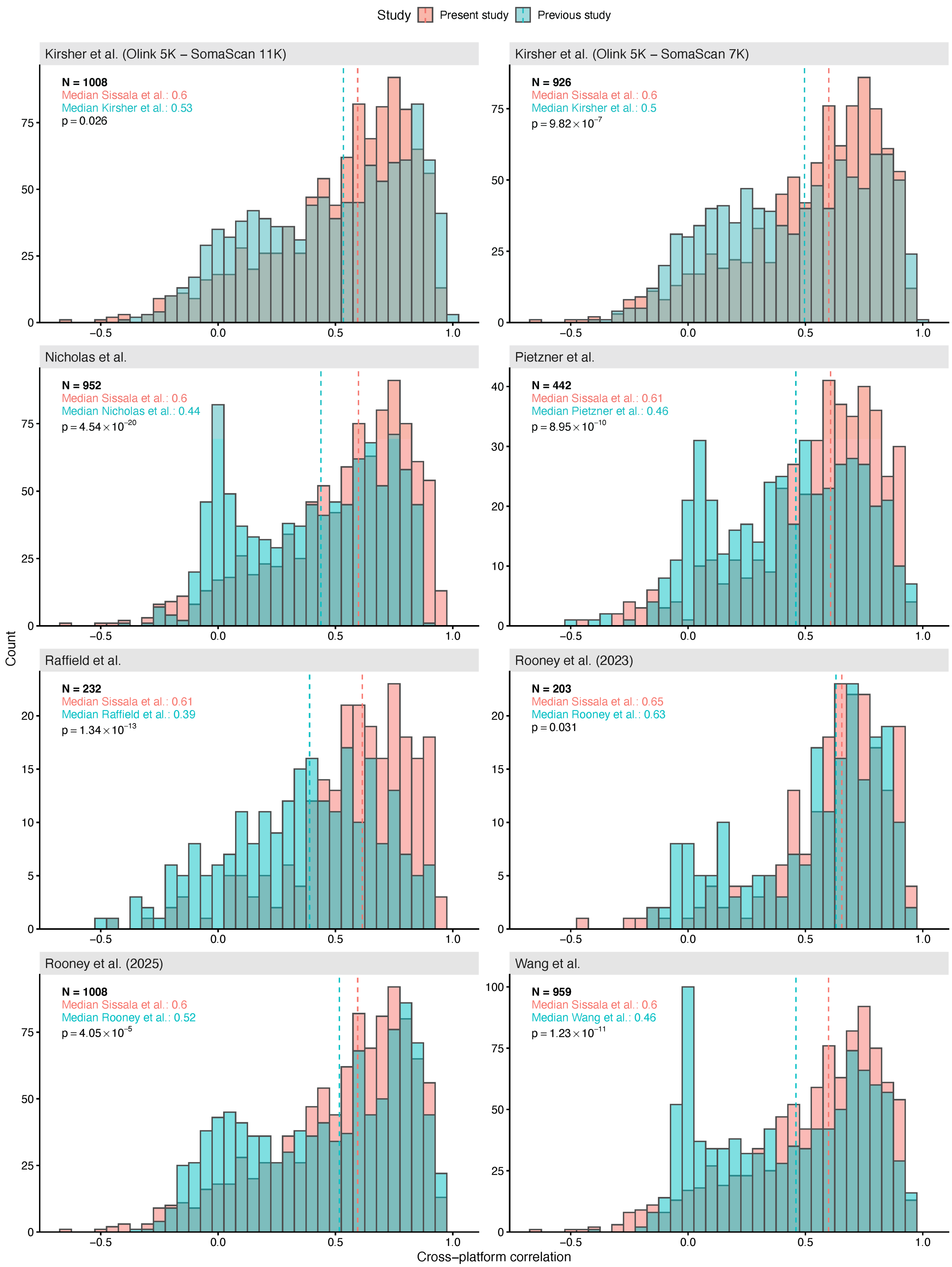

Figure legend on the next page

Figure S8. Comparison of MS-Olink correlations from the present study with Olink-SomaScan correlations from previous studies. Histograms showing per-protein MS-Olink correlations from the present study and Olink-SomaScan correlations from previous studies for matching protein pairs. The number of matching protein pairs and median cross-platform correlations for each study are shown on each plot. Dashed lines indicate the medians. P-values were determined using a two-sided Wilcoxon rank-sum test and adjusted with the FDR method. More details on the studies are given in Supplementary Data 10, and source data are provided in Supplementary Data 12. Olink 3K = Olink Explore 3072. Olink 5K = Olink Explore HT.

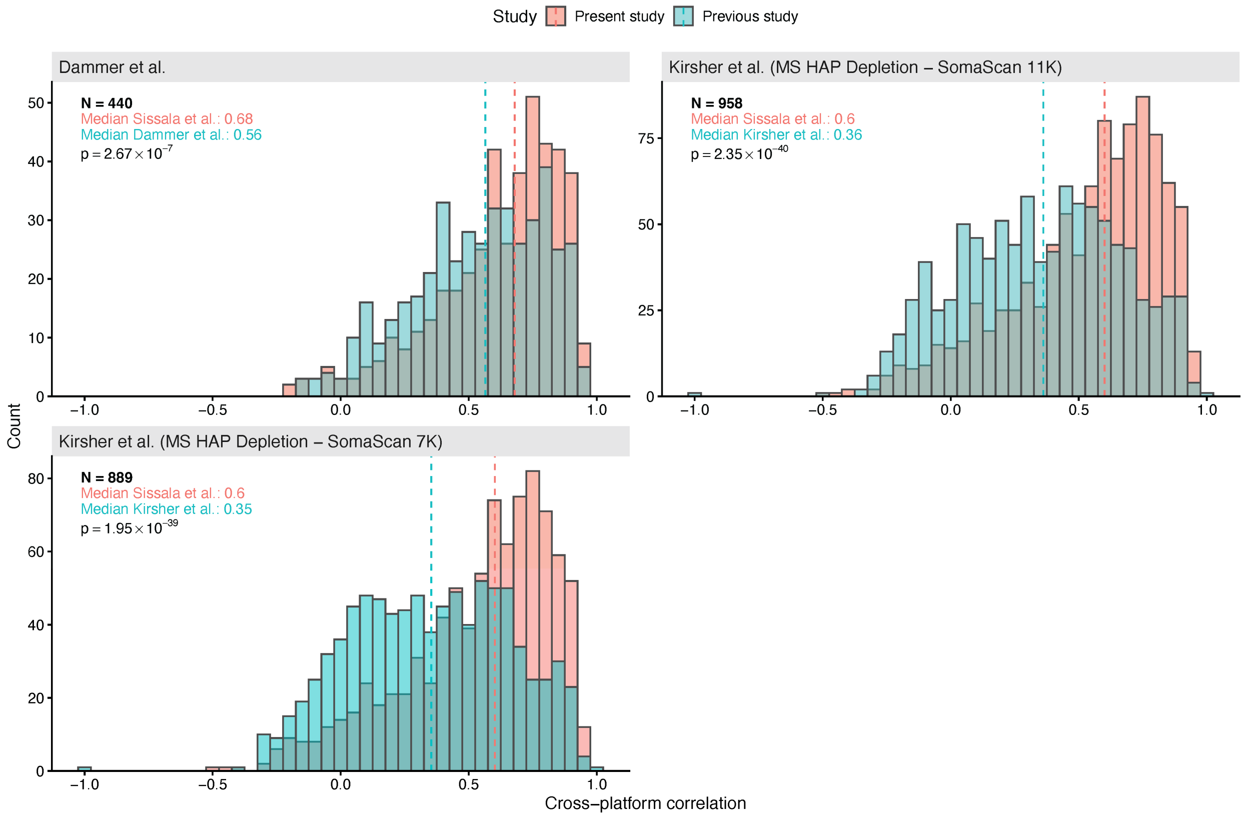

Figure S9. Comparison of MS-Olink correlations from the present study with MS-SomaScan correlations from previous studies. Histograms showing per-protein MS-Olink correlations from the present study and MS-SomaScan correlations from previous studies for matching protein pairs. The number of matching protein pairs and median cross-platform correlations for each study are shown on each plot. Dashed lines indicate the medians. P-values were determined using a two-sided Wilcoxon rank-sum test and adjusted with the FDR method. More details on the studies are given in Supplementary Data 10, and source data are provided in Supplementary Data 13. HAP Depletion = high-abundant protein depletion.

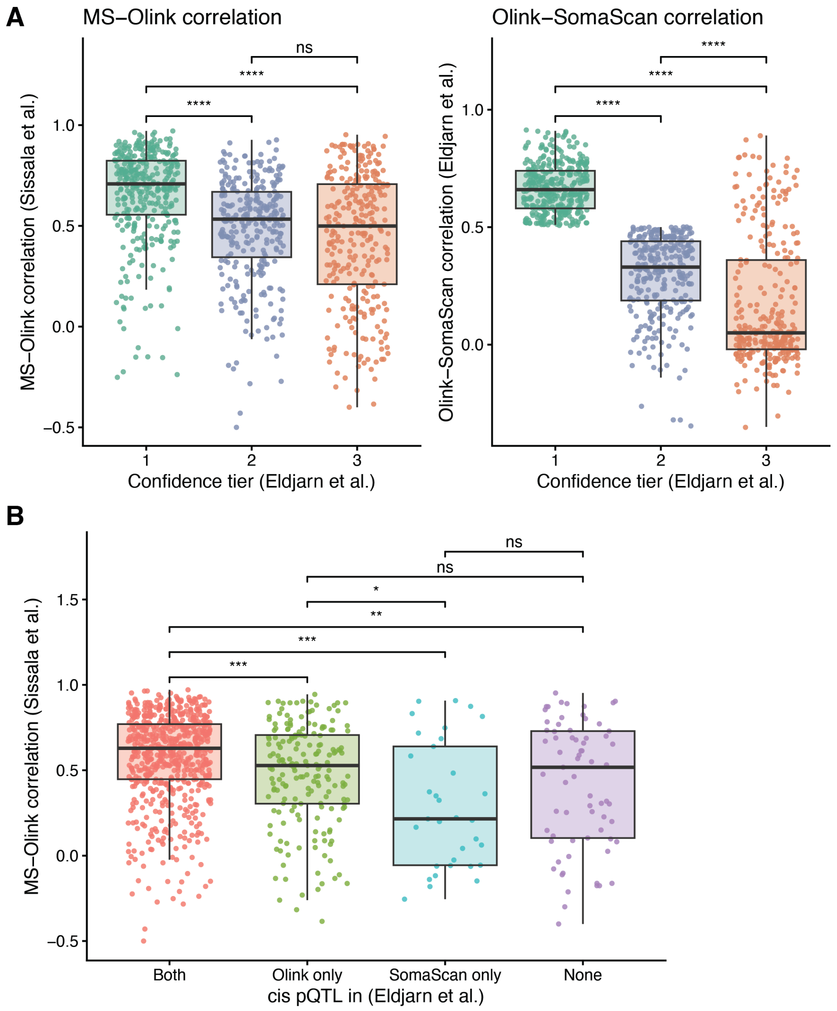

Figure S10. MS-Olink correlations by confidence of measurement with Olink and SomaScan. (A) Boxplots showing MS-Olink correlations from the current study and Olink-SomaScan correlations from Eldjarn *et al.*[1]*,* grouped by confidence tiers defined by Eldjarn *et al.* P-values were determined using a two-sided Wilcoxon rank-sum test and adjusted with the FDR method. ns = not significant, **** = p < 0.0001. **(B)** Boxplots showing MS-Olink correlations from the present study for proteins with a pQTL present on SomaScan only, Olink only, both platforms, or neither platform ("None") in Eldjarn *et al.* P-values were determined using a two-sided Wilcoxon rank-sum test and adjusted with the FDR method. ns = not significant, * = p < 0.05, ** = p < 0.01, *** = p < 0.001. Source data are provided in Supplementary Data 14.

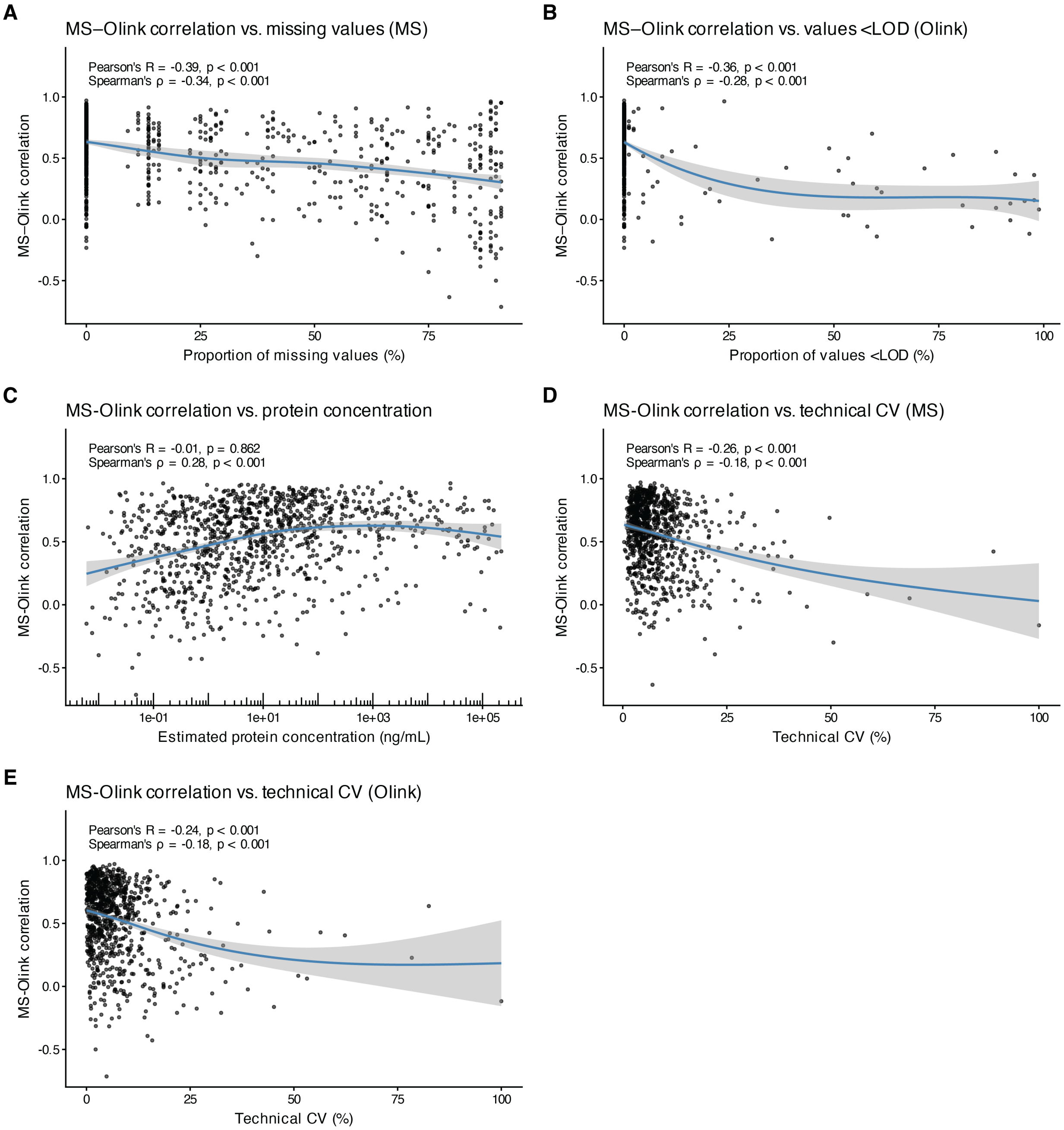

Figure S11. Relationship between technical factors shared between MS and Olink and cross-platform correlations. MS-Olink correlation versus **(A)** missing values in MS, **(B)** values <LOD in Olink, **(C)** estimated blood concentration from the HPA, and **(D-E)** technical CVs. Spearman and Pearson correlation coefficients are displayed on each plot. A nonparametric smoothing curve is shown to indicate the trend.

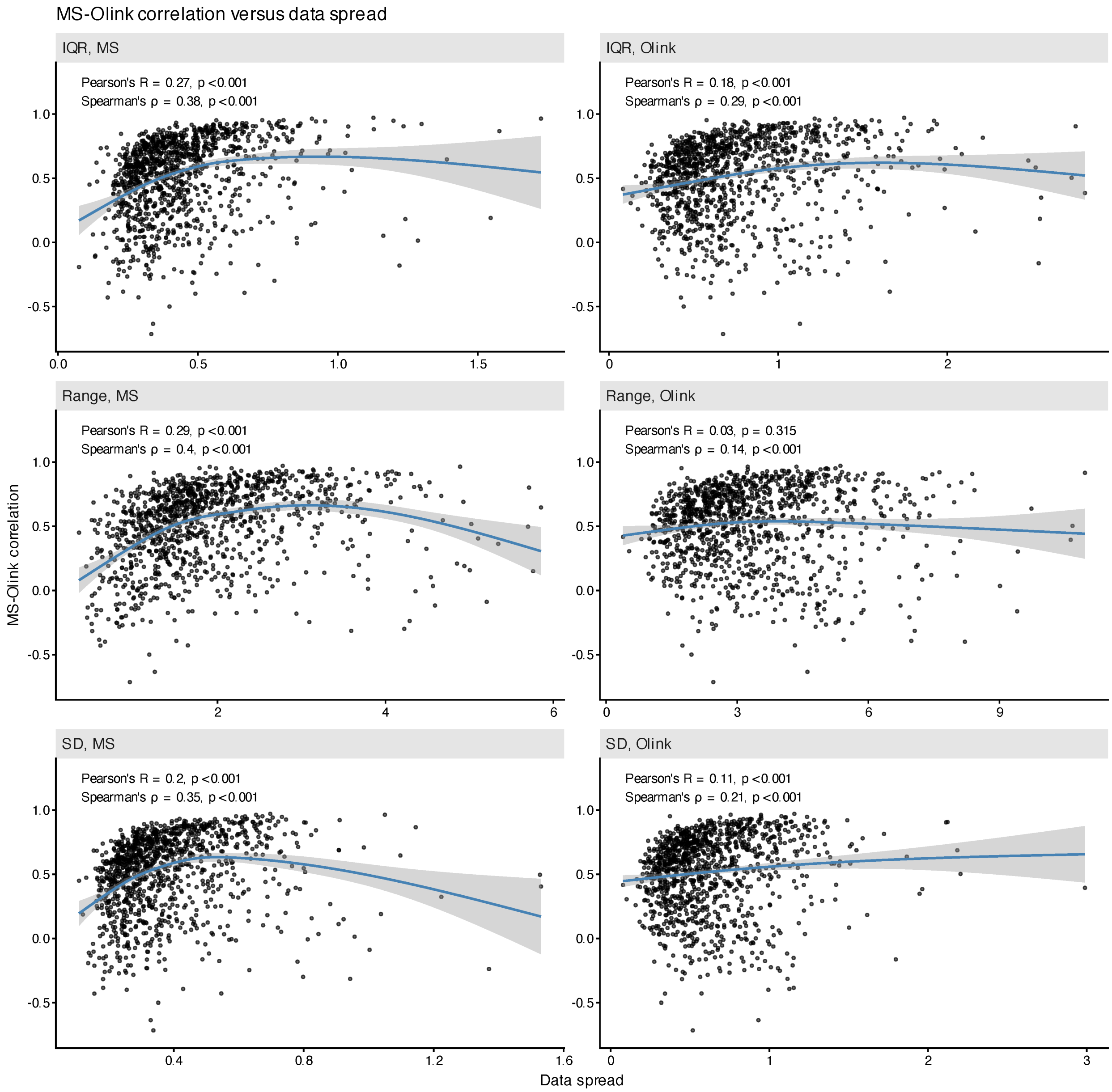

Figure S12. Relationship between data spread and cross-platform correlations. Scatter plots showing the relationship between IQR, range, and standard deviation (SD) of protein abundance values and the correlation between HiRIEF LC-MS/MS and Olink Explore 3072 measurements. Spearman and Pearson correlation coefficients are displayed on each plot. A nonparametric smoothing curve is shown to indicate the trend.

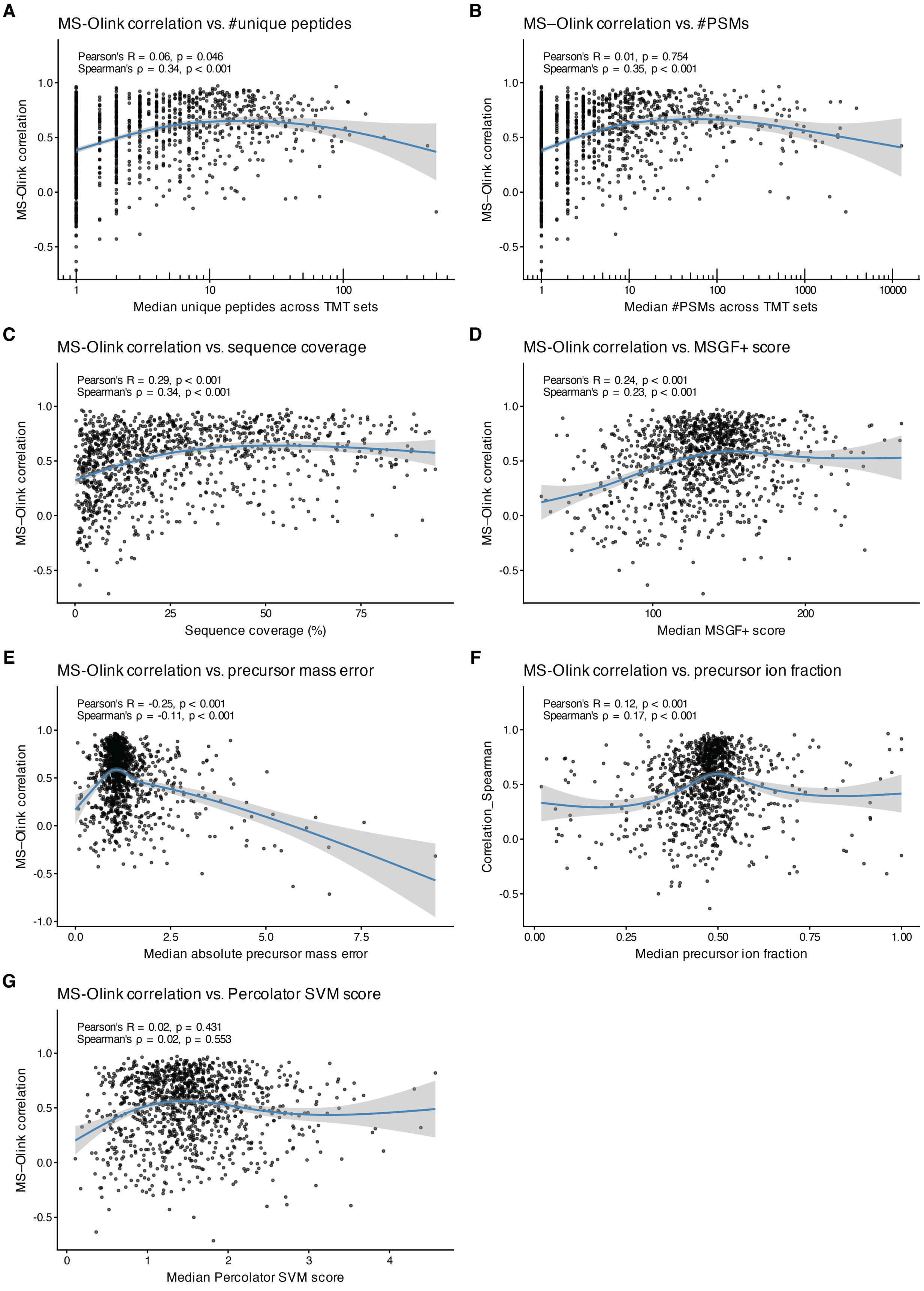
A
MS-Olink correlation vs. #unique peptides
Pearson's R = 0.06, p = 0.046
Spearman's ρ = 0.34, p < 0.001
MS-Olink correlation
Median unique peptides across TMT sets
B
MS−Olink correlation vs. #PSMs
Pearson's R = 0.01, p = 0.754
Spearman's ρ = 0.35, p < 0.001
MS−Olink correlation
Median #PSMs across TMT sets
C
MS-Olink correlation vs. sequence coverage
Pearson's R = 0.29, p < 0.001
Spearman's ρ = 0.34, p < 0.001
MS−Olink correlation
Sequence coverage (%)
D
MS-Olink correlation vs. MSGF+ score
Pearson's R = 0.24, p < 0.001
Spearman's ρ = 0.23, p < 0.001
MS−Olink correlation
Median MSGF+ score
E
MS-Olink correlation vs. precursor mass error
Pearson's R = -0.25, p < 0.001
Spearman's ρ = -0.11, p < 0.001
MS−Olink correlation
Median absolute precursor mass error
F
MS-Olink correlation vs. precursor ion fraction
Pearson's R = 0.12, p < 0.001
Spearman's ρ = 0.17, p < 0.001
Correlation_Spearman
Median precursor ion fraction
G
MS-Olink correlation vs. Percolator SVM score
Pearson's R = 0.02, p = 0.431
Spearman's ρ = 0.02, p = 0.553
MS−Olink correlation
Median Percolator SVM score

Figure S13. Relationship between MS-specific technical factors and cross-platform correlations. MS-Olink correlation versus **(A)** median (across TMT sets) number of unique peptides used for quantification, **(B)** median (across TMT sets) number of PSMs used for quantification, **(C)** protein sequence coverage, **(D)** median MS-GF+ score, **(E)** median precursor mass error, **(F)** median precursor ion fraction, and **(G)** median Percolator SVM score. The MS-GF+ score reflects the quality of the match between an observed MS/MS spectrum and a peptide—higher scores indicate better matches[2]. The Percolator SVM score reflects the confidence of PSMs, with higher scores indicating more confident PSMs[3]. The x-axes in **(A)** and **(B)** are on a $\log_{10}$ scale. Spearman and Pearson correlation coefficients are shown on each plot, with a nonparametric smoothing curve indicating the trend.

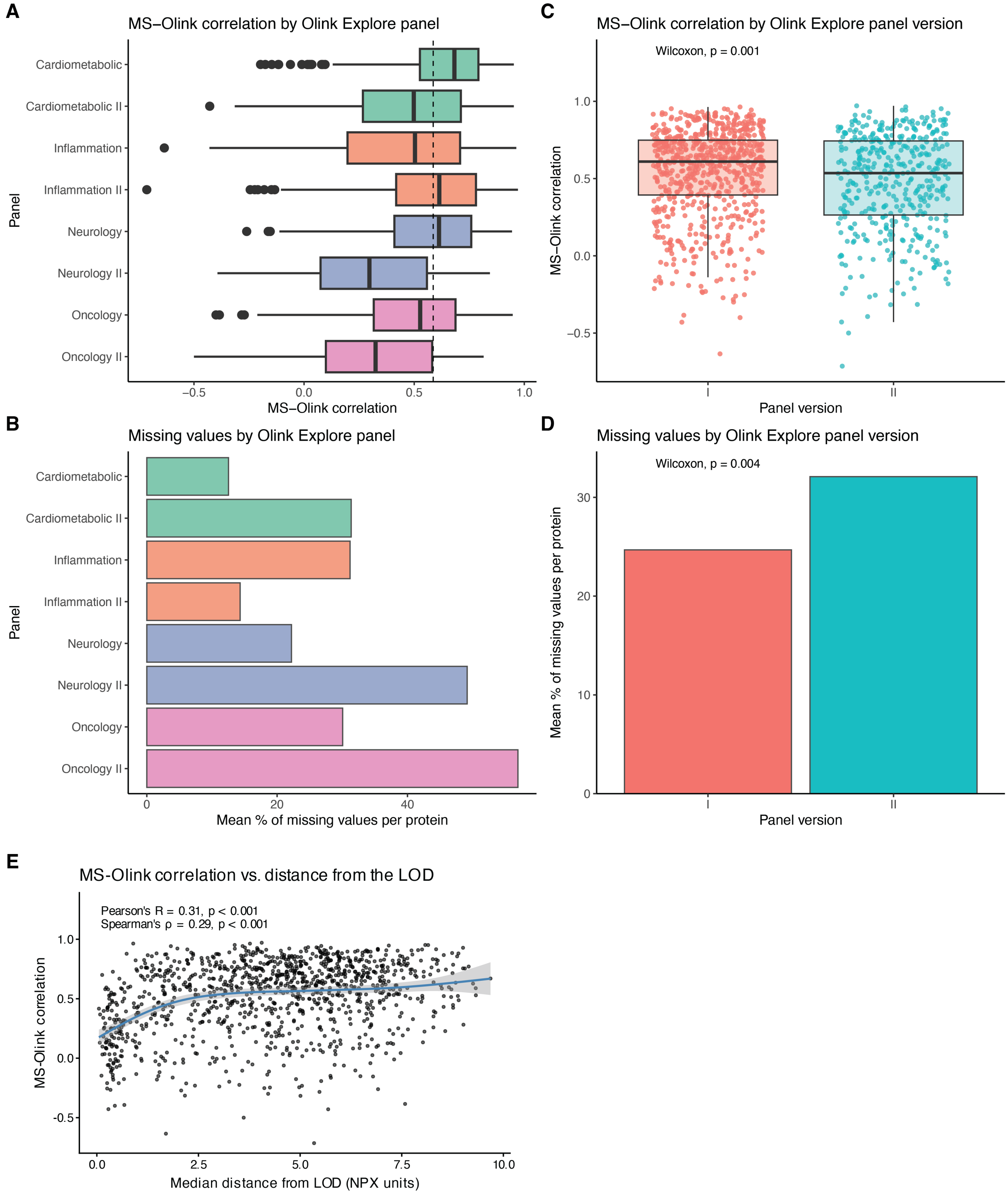

Figure S14. Relationship between Olink-specific technical factors and cross-platform correlations. (A) MS-Olink correlations by Olink Explore panel. The dashed line indicates the median correlation across all proteins. **(B)** Mean proportion of missing values (= values <LOD) per protein by Olink Explore panel. **(C)** MS-Olink correlations by Olink Explore panel version. Version I panels were also included in the earlier version of the Explore platform, the Olink Explore

1536, while version II panels were new to the Olink Explore 3072. **(D)** Mean proportion of missing values per protein by Olink Explore panel version. **(E)** MS-Olink correlation versus median distance of NPX values from LOD. Spearman and Pearson correlation coefficients are shown on the plot, with a nonparametric smoothing curve indicating the trend.

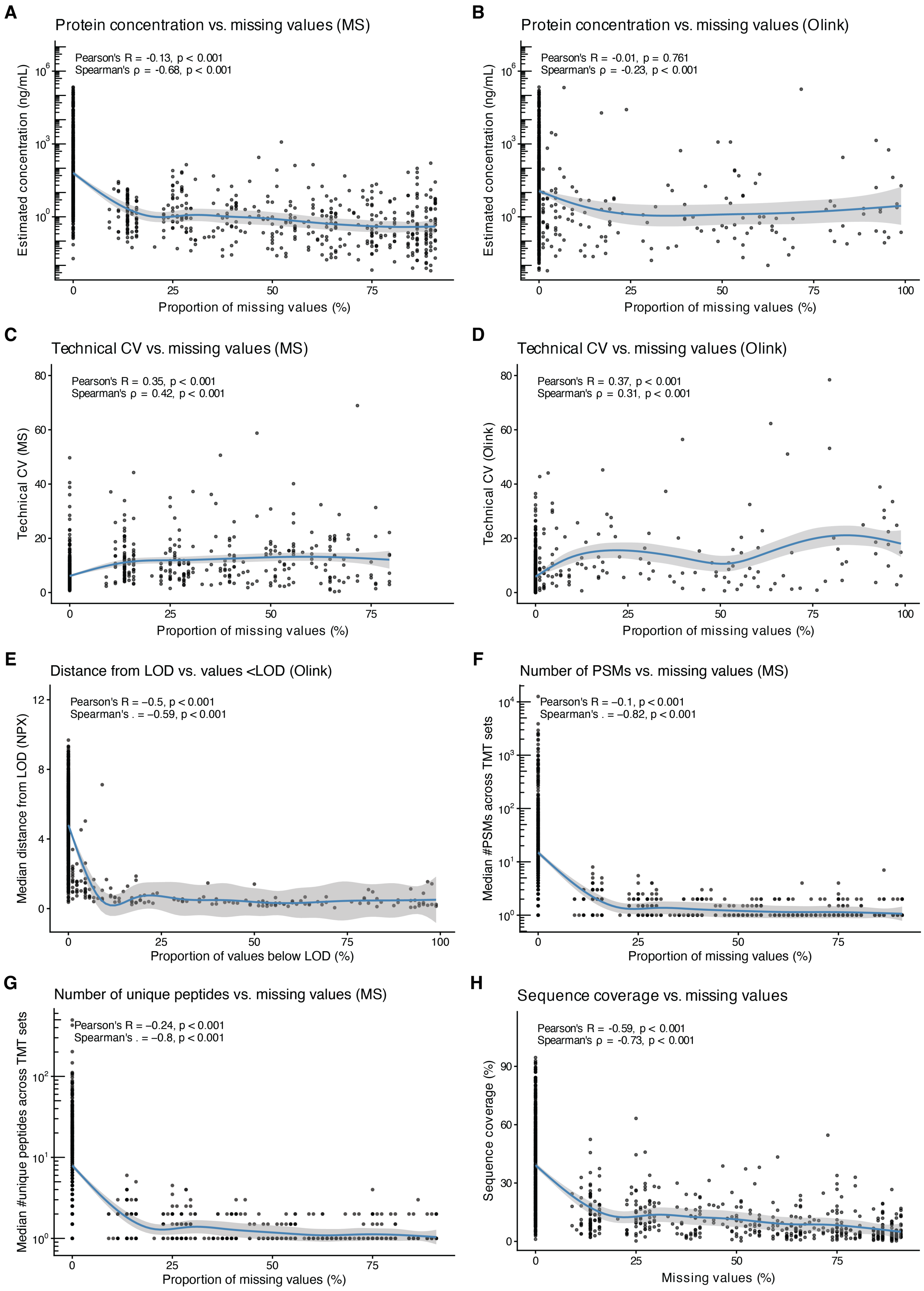
A
Protein concentration vs. missing values (MS)
Pearson's R = -0.13, p < 0.001
Spearman's ρ = -0.68, p < 0.001
Estimated concentration (ng/mL)
Proportion of missing values (%)
B
Protein concentration vs. missing values (Olink)
Pearson's R = -0.01, p = 0.761
Spearman's ρ = -0.23, p < 0.001
Estimated concentration (ng/mL)
Proportion of missing values (%)
C
Technical CV vs. missing values (MS)
Pearson's R = 0.35, p < 0.001
Spearman's ρ = 0.42, p < 0.001
Technical CV (MS)
Proportion of missing values (%)
D
Technical CV vs. missing values (Olink)
Pearson's R = 0.37, p < 0.001
Spearman's ρ = 0.31, p < 0.001
Technical CV (Olink)
Proportion of missing values (%)
E
Distance from LOD vs. values <LOD (Olink)
Pearson's R = −0.5, p < 0.001
Spearman's . = −0.59, p < 0.001
Median distance from LOD (NPX)
Proportion of values below LOD (%)
F
Number of PSMs vs. missing values (MS)
Pearson's R = −0.1, p < 0.001
Spearman's . = −0.82, p < 0.001
Median #PSMs across TMT sets
Proportion of missing values (%)
G
Number of unique peptides vs. missing values (MS)
Pearson's R = −0.24, p < 0.001
Spearman's . = −0.8, p < 0.001
Median #unique peptides across TMT sets
Proportion of missing values (%)
H
Sequence coverage vs. missing values
Pearson's R = -0.59, p < 0.001
Spearman's ρ = -0.73, p < 0.001
Sequence coverage (%)
Missing values (%)

Figure S15. Relationship between missing values and other technical factors. (A-B) Estimated blood concentration from the HPA versus proportion of missing values per protein in **(A)** HiRIEF LC-MS/MS and **(B)** Olink Explore 3072 data. The y-axes are presented on a log_{10}-scale. **(C-D)** Technical CVs versus proportion of missing values per protein in **(C)** MS and **(D)** Olink. **(E)** Median distance of NPX values from LOD versus the proportion of missing values per protein in Olink data. **(F-G)** Median number of **(F)** PSMs and **(G)** unique peptides used for quantification across TMT sets versus the proportion of missing values per protein in MS data. The y-axes are on a log_{10} scale **(H)** Protein sequence coverage by MS versus proportion of missing values in MS data. Spearman and Pearson correlation coefficients are shown on each plot, with a nonparametric smoothing curve indicating the trend.

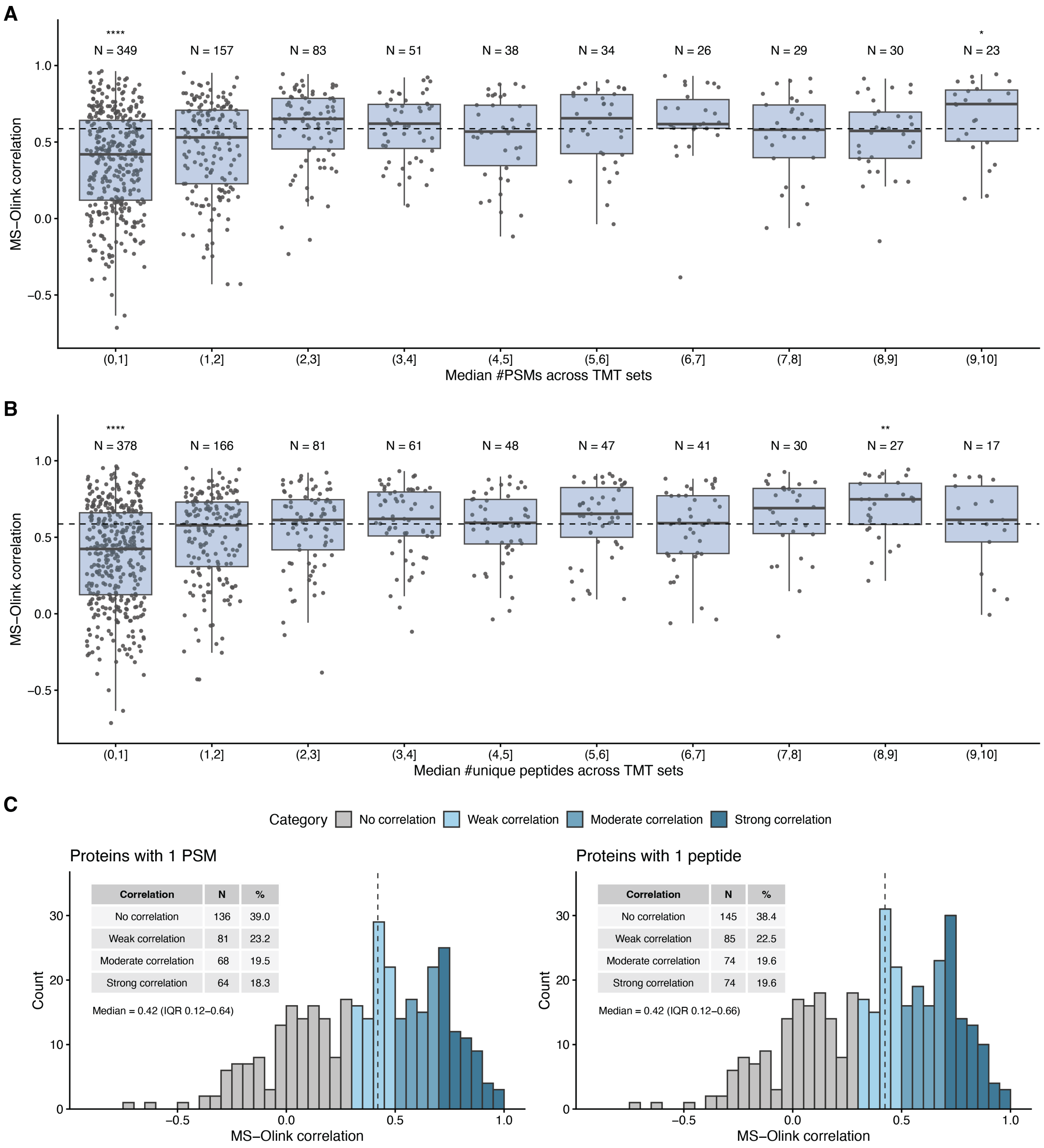

Figure S16. Impact of the number of PSMs and peptides on cross-platform correlations. **(A)** Boxplots showing the distribution of MS-Olink correlations by the median number of PSMs per protein in the MS data. Asterisks indicate statistically significant differences from the median MS-Olink correlation of all overlapping proteins, shown as a dashed line. P-values were determined using a two-sided Wilcoxon rank-sum test and adjusted for multiple testing with the FDR method. * = $p < 0.05$, **** = $p < 0.0001$. **(B)** Same as (A), but for the number of unique peptides per protein. ** = $p < 0.01$, **** = $p < 0.0001$. **(C)** Histograms of MS-Olink correlations for proteins with one median PSM across TMT sets (left) and proteins with one median unique peptide across TMT sets (right). Dashed lines indicate medians. Correlations were categorized

as no correlation: $\rho \in [-1, 0.3)$; weak correlation: $\rho \in [0.3, 0.5)$; moderate correlation: $\rho \in [0.5, 0.7)$; and strong correlation: $\rho \in [0.7, 1.0]$.

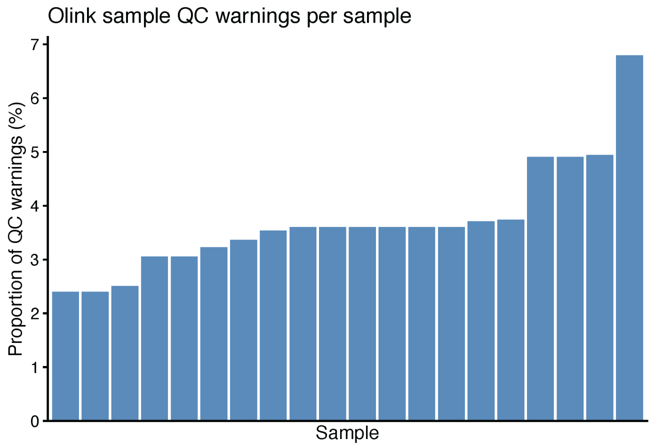

Figure S17. Sample QC warnings in the Olink data. Proportion of NPX values flagged with a sample QC warning, shown or every sample that had at least one sample QC warning in the Olink Explore 3072 data.

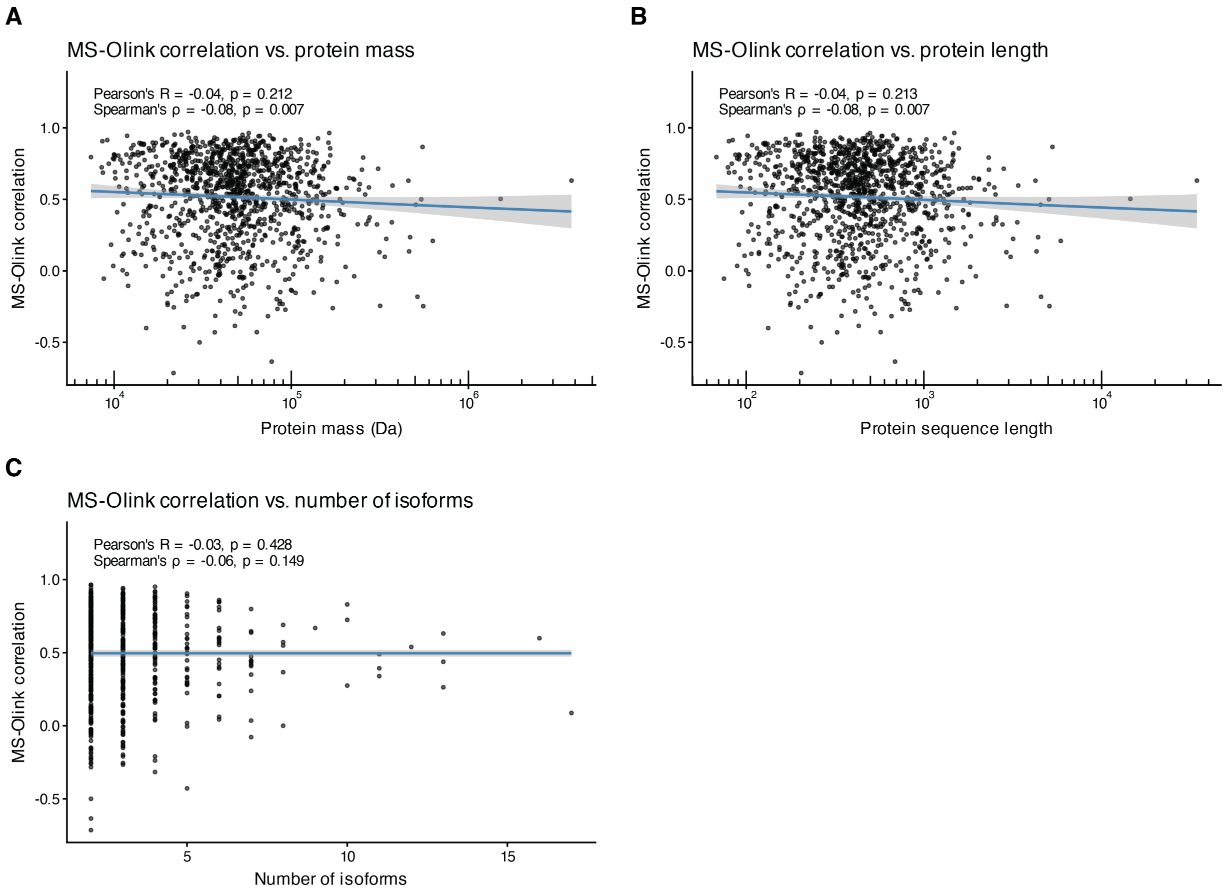

Figure S18. Relationship between protein properties and cross-platform correlations. MS-Olink correlation versus **(A)** protein mass, **(B)** protein sequence length, and **(C)** the number of isoforms reported in UniProt. Spearman and Pearson correlation coefficients are shown on each plot, with a nonparametric smoothing curve indicating the trend.

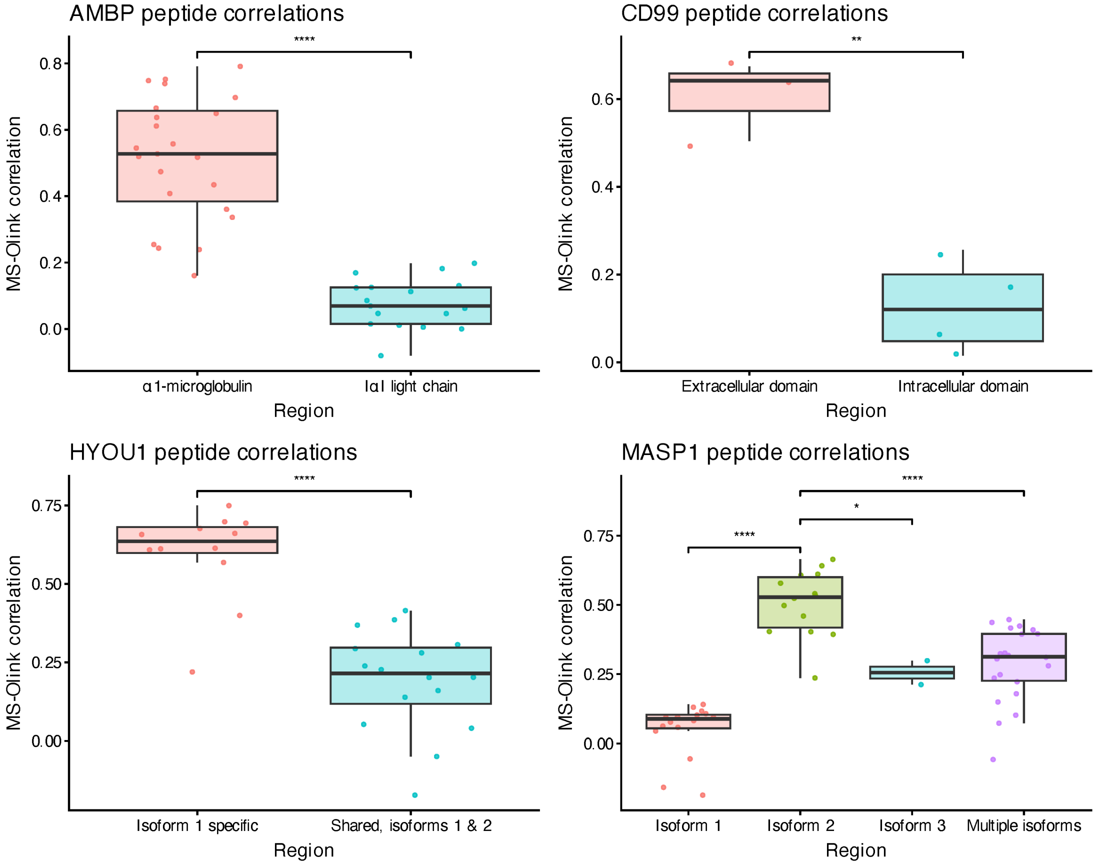

Figure S19. Peptide-Olink correlations across different protein sequence regions for select examples. Boxplots showing the MS-Olink correlation at the peptide level for different regions of the AMBP, CD99, HYOU1, and MASP1 protein sequences. For AMBP, correlations are shown for peptides mapping to the α1-Microglobulin (residues 20-203) and Inter-α-Trypsin Inhibitor Light Chain (IαI) regions (residues 206-352). For CD99, correlations are shown for peptides mapping to the extracellular (residues 23-122) and intracellular domains (residues 148-185). For HYOU1, correlations are shown for peptides mapping to regions shared by isoforms 1 and 2 (residues 88-602) and the region unique to isoform 1 (residues 647-999). For MASP1, correlations are presented for peptides mapping uniquely to each isoform (UniProt IDs: P48740-1, P48740-2, and P48740-3), and peptides mapping to multiple isoforms ("Multiple isoforms"). P-values were determined using two-sided Welch's t-tests and adjusted using the FDR method. ns = not significant, * = $p < 0.05$, ** = $p < 0.01$, *** = $p < 0.001$, **** = $p < 0.0001$.

		TMT set							
		**setA**	**setB**	**setC**	**setD**	**setE**	**setF**	**setG**	**setH**
**TMT tag**	**126**	**IS**	S026	S015	S002	S009	S078	S031	S007
	**127N**	**S049a**	S003	S022	S090	S029	S087	S017	S112
	**127C**	S101	**IS**	**S049b**	S021	S012	S057	S054	S023
	**128N**	S065	S041	S096	**S089b**	S103	S066	S052	S075
	**128C**	S025	S102	S008	S070	**S034b**	S079	S032	S073
	**129N**	S074	S027	**IS**	S082	S056	**S058b**	S011	S097
	**129C**	S100	S036	S094	S010	S085	S059	**S071b**	S086
	**130N**	S018	S005	S068	**IS**	S035	S106	S061	**S098b**
	**130C**	S114	S084	S108	S040	S055	S064	S081	S077
	**131N**	S047	**S034a**	S069	S093	**IS**	S095	S104	S092
	**131C**	S024	**S098a**	S004	S033	S016	S013	S001	S044
	**132N**	**S071a**	**S058a**	S050	S110	S113	**IS**	S062	S053
	**132C**	S109	S042	S107	S039	S028	S038	S014	S091
	**133N**	**S089a**	S105	S099	S080	S063	S083	**IS**	S076
	**133C**	S037	S030	S006	S045	S046	S019	S060	S051
	**134N**	S020	S043	S072	S088	S067	S048	S111	**IS**

Table S1. TMT labeling scheme. Assignment of samples to TMT sets and individual TMTs for the HiRIEF LC-MS/MS analysis. Duplicate samples are highlighted and labeled with "a" and "b". "IS" indicates the internal standard sample used for normalization across TMT batches.

Fraction	Gradient length
fraction_01	accumulate in trap column
fraction_02	50 min
fraction_03	70 min
fraction_04	70 min
fraction_05	90 min
fraction_06	90 min
fraction_07	110 min
fraction_08	110 min
fraction_09	110 min
fraction_10	110 min
fraction_11	110 min
fraction_12	110 min
fraction_13	110 min
fraction_14	110 min
fraction_15	90 min
fraction_16	70 min
fraction_17	70 min
fraction_18	50 min
fraction_19	50 min
fraction_20	accumulate in trap column
fraction_21	accumulate in trap column
fraction_22	accumulate in trap column
fraction_23	accumulate in trap column
fraction_24	accumulate in trap column
fraction_25	accumulate in trap column
fraction_26	accumulate in trap column
fraction_27	50 min
fraction_28	50 min
fraction_29	70 min
fraction_30	70 min
fraction_31	70 min
fraction_32	70 min
fraction_33	70 min
fraction_34	70 min
fraction_35	70 min
fraction_36	70 min
fraction_37	70 min
fraction_38	accumulate in trap column

fraction_39	accumulate in trap column
fraction_40	accumulate in trap column
fraction_41	accumulate in trap column
fraction_42	accumulate in trap column
fraction_43	accumulate in trap column
fraction_44	accumulate in trap column
fraction_45	70 min
fraction_46	70 min
fraction_47	70 min
fraction_48	70 min
fraction_49	70 min
fraction_50	50 min
fraction_51	50 min
fraction_52	accumulate in trap column
fraction_53	accumulate in trap column
fraction_54	accumulate in trap column
fraction_55	70 min
fraction_56	50 min
fraction_57	50 min
fraction_58	accumulate in trap column
fraction_59	accumulate in trap column
fraction_60	accumulate in trap column
fraction_61	accumulate in trap column
fraction_62	accumulate in trap column
fraction_63	accumulate in trap column
fraction_64	accumulate in trap column
fraction_65	accumulate in trap column
fraction_66	accumulate in trap column
fraction_67	accumulate in trap column
fraction_68	accumulate in trap column
fraction_69	accumulate in trap column
fraction_70	accumulate in trap column
fraction_71	accumulate in trap column
fraction_72	50 min

Table S2. Gradient length for HiRIEF fractions. Lists each high-resolution isoelectric focusing (HiRIEF) fraction analyzed by liquid chromatography tandem mass spectrometry (LC-MS/MS) and the corresponding gradient length used during peptide separation by LC.

Supplementary references

1. Eldjarn, G. H. *et al.* Large-scale plasma proteomics comparisons through genetics and disease associations. *Nature* **622**, 348–358 (2023).
2. Kim, S. & Pevzner, P. A. MS-GF+ makes progress towards a universal database search tool for proteomics. *Nat. Commun.* **5**, 5277 (2014).
3. Granholm, V. *et al.* Fast and Accurate Database Searches with MS-GF+Percolator. *J. Proteome Res.* **13**, 890–897 (2014).
