# Supplementary material for: Comparative evaluation of Olink Explore 3072 and mass spectrometry with peptide fractionation for plasma proteomics
Source: Commun Chem. 2025 Nov 4;8:327. doi: 10.1038/s42004-025-01753-2 (PMC12586489; doi:10.1038/s42004-025-01753-2)
Supplement: Supplementary file 3 — Description of Additional Supplementary Files [file 42004_2025_1753_MOESM3_ESM.pdf]

Description of Additional Supplementary Files

File name: Supplementary Data 1

Description: List of proteins measured and detected with HiRIEF LCMS/MS and/or Olink Explore 3072. The table includes protein identifiers (UniProt ID, gene name, description), Olink assay metadata (Olink ID, assay name, panel, panel lot number, and plate ID), missing value frequencies ("Perc.Missing.MS" and "Perc.Missing.Olink"), overlap between platforms ("Overlapping"), overlap with the Human Protein Atlas (HPA, "In.HPA", "In.HPA.Plasma") and overlap with the Human Plasma Proteome Project (HPPP, "In.HPPP"). For Olink, "Perc.Missing" refers to the percentage of values below the limit of detection (LOD). The HPA plasma proteins were defined as proteins with a blood concentration in the HPA or proteins annotated as "Secreted to blood". The reference plasma proteome ("In.Ref.Plasma.Proteome") was compiled by combining the HPA plasma proteins and proteins from the HPPP.

File name: Supplementary Data 2

Description: Overrepresentation analysis (ORA) of protein annotations from the Human Protein Atlas (HPA). Frequency and ORA of HPA annotations among proteins detected with HiRIEF LC-MS/MS and Olink Explore 3072. Columns include annotation category ("HPA.category"), specific annotation ("Annotation"), background counts and frequencies ("N_BG", "Freq_BG"), counts and frequencies among MS or Olink protein lists ("N_MS", "Freq_MS", "N_Olink", "Freq_Olink"), and differences between platforms ("N.diff", "Freq.diff"). Statistical significance of differences in frequencies was assessed using Fisher's exact test ("Fisher.pvalue" and "Fisher.adj.p"). ORA was performed using a hypergeometric test, with all proteins detected by MS and/or Olink used as the background ("Enr.pvalue", "Enr.adj.p", and "Enriched.platform").

File name: Supplementary Data 3

Description: Overrepresentation analysis (ORA) of Gene Ontology (GO) terms. ORA of GO terms among proteins detected with HiRIEF LC-MS/MS and Olink Explore 3072. Results include the number and proportion of detected proteins associated with each GO term ("Count", "GeneRatio"), and the corresponding proportion in the background protein list ("BgRatio"). Fold enrichment ("FoldEnrichment") was calculated as GeneRatio/BgRatio. Additional enrichment metrics include RichFactor

(Count/background count) and z-score. Statistical significance is reported in columns "p.value", "p.adjust", and "qvalue".

File name: Supplementary Data 4

Description: Coverage of FDAapproved plasma protein biomarkers. Comparison of FDA-approved plasma protein biomarkers, based on a list compiled by Anderson32, detected with HiRIEF LCMS/MS and Olink Explore 3072. The table includes protein names and IDs from the FDA list ("Protein.name", "UniProt_FDA"), and detection status and protein IDs for MS and Olink.

File name: Supplementary Data 5

Description: Technical coefficients of variation (CVs). Technical CVs calculated from repeated measurements of proteins measured by HiRIEF LC-MS/MS and Olink Explore 3072. The table includes protein IDs, Olink assay details, the technical CV for each protein, and an indicator of whether a technical CV could be calculated for the protein ("Analyzed").

File name: Supplementary Data 6

Description: Differential abundance analysis (DAA) of overlapping proteins. DAA was performed to compare protein levels between females (N=37) and males (N=51) in the HiRIEF LC-MS/MS and Olink Explore 3072 datasets for all overlapping proteins (N = 1129). Results include the difference in protein levels between groups (log2-fold change, "log2FC"), mean protein levels in each group ("mean.testGrp", "mean.refGrp"), group sample sizes ("n.testGrp", "n.refGrp"), total sample size ("n.total"), test statistics, confidence intervals, and p-values. Statistical significance is indicated in the "Significance" columns, with "S" = significant and "NS" = not significant. Males were treated as the reference group (refGrp), and females as the test group (testGrp).

File name: Supplementary Data 7

Description: Differential abundance analysis (DAA) of overlapping proteins with no missing values. DAA was performed to compare protein levels between females (N=37) and males (N=51) in the HiRIEF LC-MS/MS and Olink datasets for overlapping proteins with no missing values (N = 569). Results include the difference in protein levels between groups (log2-fold change, "log2FC"), mean protein levels in each group ("mean.testGrp", "mean.refGrp"), group

sample sizes ("n.testGrp", "n.refGrp"), total sample size ("n.total"), test statistics, confidence intervals, and p-values. Statistical significance is indicated in the "Significance" columns, with "S" = significant and "NS" = not significant. Males were treated as the reference group (refGrp), and females as the test group (testGrp).

File name: Supplementary Data 8

Description: Cross-platform correlations and technical factors. Correlations between HiRIEF LC-MS/MS and Olink Explore 3072 protein measurements. Correlations were calculated both for Olink data with <LOD values and QC warnings retained, and for Olink data with <LOD values and QC warnings set to missing (columns marked with “cleanData”). The table includes protein identifiers (UniProt ID, gene name, description), Olink assay metadata (Olink ID, assay name, panel), Pearson and Spearman correlations between measurements, and sample size used to calculate correlations ("N" and "N.cleanData"). Columns from Z onward contain data on various technical factors, such as missing value proportions, number of peptide spectrum matches (PSMs) and unique peptides per protein in MS data, number of sample QC warnings per protein in Olink data, and data spread (CV, IQR, SD, Range), among others. Abbreviations: N = number; QC = quality control; Abs = absolute; IQR = interquartile range; SD = standard deviation; CV = coefficient of variation.

File name: Supplementary Data 9

Description: Cross-platform correlations for protein isoforms. Spearman correlations between HiRIEF LC-MS/MS and Olink Explore 3072 measurements for protein isoforms—proteins with matching gene names but differing UniProt IDs between MS and Olink data.

File name: Supplementary Data 10

Description: Summary of studies included in the comparison of crossplatform correlations. This table summarizes key information about each study included in the comparison of crossplatform correlations between studies. It reports the total number of overlapping samples and proteins between platforms in each study, as well as the cross-platform correlation for these proteins. It further specifies how proteins were matched between platforms and to the present study ("Matching to present study by"), the number of proteins overlapping with the present study, and the median crossplatform correlation for these proteins. It also details which MS, Olink, and SomaLogic platforms were used, and how data were pre-processed. Abbreviations: TMT = tandem mass tag; LOD = limit of detection; QC = quality control; CV = coefficient of variation.

File name: Supplementary Data 11

Description: Comparison to MSOlink correlations from previous studies. Comparison of cross-platform correlations between HiRIEF LC-MS/MS and Olink Explore 3072 with correlations reported between MS and Olink platforms in previous studies. The table includes protein identifiers, Olink assay identifiers ("OlinkID"), MS-Olink correlations, sample sizes ("N"), and pvalues from the present study (columns marked with "Sissala") and each previous study analyzed (columns marked with "Previous.study").

File name: Supplementary Data 12

Description: Comparison to Olink-SomaScan correlations from previous studies. Comparison of cross-platform correlations between HiRIEF LC-MS/MS and Olink Explore 3072 with correlations reported between Olink and SomaScan platforms in previous studies. The table includes protein identifiers, Olink assay identifiers ("OlinkID"), cross-platform correlations, sample sizes ("N"), and pvalues from the present study (columns marked "Sissala") and previous studies (columns marked "Previous.study"). SomaScan assay identifiers from each previous study ("SomaID", "SomaScan.SeqID") are also included, when available.

File name: Supplementary Data 13

Description: Comparison to MSSomaScan correlations from previous studies. Comparison of cross-platform correlations between HiRIEF LC-MS/MS and Olink Explore 3072 with correlations reported between MS and SomaScan platforms in previous studies. The table contains protein identifiers, cross-platform correlations, sample sizes ("N"), and pvalues from the present study (columns marked "Sissala") and previous studies (columns marked "Previous.study"). Olink assay identifiers from the present study ("OlinkID") and SomaScan assay identifiers from each previous study ("SomaID", "SomaScan.SeqID") are included when available.

File name: Supplementary Data 14

Description: Cross-platform correlations by confidence tier. Crossplatform correlations between HiRIEF LCMS/MS and Olink Explore 3072, shown alongside Olink-SomaScan correlations and confidence tier classifications from Eldjarn et al.14. Confidence tiers are coded from 1 to 3, with 1 representing the highest confidence. The columns "cis.pQTL.olink_Eldjarn" and "cis.pQTL.soma_Eldjarn" indicate whether a protein had a cis-pQTL detected using Olink or SomaScan, respectively, in Eldjarn et al.14 (Y = yes; N = no). Proteins quantified with high

confidence in both studies (defined as having cross-platform correlation ≥0.7 in both) are indicated in the column "High.corr.in.both.studies".

File name: Supplementary Data 15

Description: Results from univariable linear regression analyses assessing the association between technical factors and MS-Olink correlation. Each technical factor was used as an independent variable in a separate linear regression model, with the Spearman correlation between HiRIEF LC-MS/MS and Olink Explore 3072 protein measurements as the dependent variable. The table includes regression coefficients ("Coef"), p-values, proportion of variance explained (R squared), and other model statistics ("Conf.low", "Conf.high" = 95% confidence interval bounds; "Std.error" = Standard error; "Statistic" = t-statistic from the regression).

File name: Supplementary Data 16

Description: Gene Set Enrichment Analysis (GSEA) on crossplatform correlations. GSEA was performed to assess whether specific Human Protein Atlas (HPA) annotations were enriched among proteins with particularly high or low correlations between HiRIEF LC-MS/MS and Olink Explore 3072 measurements. Results include the number of proteins associated with each HPA annotation ("setSize"), enrichment scores (NES = normalized enrichment score), and statistical significance. The "leading_edge" column contains leading edge analysis results (see the GSEA User manual for details https://www.gseamsigdb.org/gsea/doc/GSEAUserGuideFrame .html). The "core_enrichment" and "Gene.Name" columns contain the UniProt IDs and gene names of proteins in the leading-edge subset, i.e. proteins driving the enrichment. The "Rank" column indicates the position in the ranked list of correlations at which the enrichment score peaks.

File name: Supplementary Data 17

Description: Overrepresentation analysis (ORA) on cross-platform correlations. ORA was conducted to identify Gene Ontology (GO) terms and Human Protein Atlas (HPA) annotations significantly enriched among proteins with low (Spearman's correlation < 0.3) or high (correlation ≥ 0.7) crossplatform correlations between HiRIEF LCMS/MS and Olink Explore 3072. Results include the number and proportion of proteins of the low/high correlation categories associated with each GO term ("Count", "GeneRatio"), and the corresponding proportion in the background protein list ("BgRatio"). Fold enrichment ("FoldEnrichment") was calculated as GeneRatio/BgRatio. Additional enrichment metrics include RichFactor (Count/background count) and z-score. Statistical significance is reported in columns "p.value", "p.adjust", and "qvalue".

File name: Supplementary Data 18

Description: Cross-platform correlations at the peptide level. Spearman correlations between peptides quantified by HiRIEF LC-MS/MS and the corresponding Olink Explore 3072 assays. The table includes peptide identifiers ("Peptide.label"), peptide sequences, protein identifiers in MS and Olink data, Olink assay identifiers (Olink ID and assay name), correlation coefficients, and sample sizes ("N") used for correlation calculations.

File name: Supplementary Data 19

Description: Source Data
