# Supplementary material for: Comparative evaluation of Olink Explore 3072 and mass spectrometry with peptide fractionation for plasma proteomics
Source: Commun Chem. 2025 Nov 4;8:327. doi: 10.1038/s42004-025-01753-2 (PMC12586489; doi:10.1038/s42004-025-01753-2)
Supplement: Supplementary file 23 — Reporting Summary [file 42004_2025_1753_MOESM23_ESM.pdf]

nature portfolio

Corresponding author(s): Noora Sissala, Maria Pernemalm

Last updated by author(s): Sep 29, 2025

Reporting Summary

Nature Portfolio wishes to improve the reproducibility of the work that we publish. This form provides structure for consistency and transparency in reporting. For further information on Nature Portfolio policies, see our Editorial Policies and the Editorial Policy Checklist.

Statistics

For all statistical analyses, confirm that the following items are present in the figure legend, table legend, main text, or Methods section.

n/a	Confirmed	
☐	☒	The exact sample size (*n*) for each experimental group/condition, given as a discrete number and unit of measurement
☐	☒	A statement on whether measurements were taken from distinct samples or whether the same sample was measured repeatedly
☐	☒	The statistical test(s) used AND whether they are one- or two-sided<br>*Only common tests should be described solely by name; describe more complex techniques in the Methods section.*
☒	☐	A description of all covariates tested
☐	☒	A description of any assumptions or corrections, such as tests of normality and adjustment for multiple comparisons
☐	☒	A full description of the statistical parameters including central tendency (e.g. means) or other basic estimates (e.g. regression coefficient) AND variation (e.g. standard deviation) or associated estimates of uncertainty (e.g. confidence intervals)
☐	☒	For null hypothesis testing, the test statistic (e.g. *F*, *t*, *r*) with confidence intervals, effect sizes, degrees of freedom and *P* value noted<br>*Give P values as exact values whenever suitable.*
☒	☐	For Bayesian analysis, information on the choice of priors and Markov chain Monte Carlo settings
☒	☐	For hierarchical and complex designs, identification of the appropriate level for tests and full reporting of outcomes
☐	☒	Estimates of effect sizes (e.g. Cohen's *d*, Pearson's *r*), indicating how they were calculated

Our web collection on statistics for biologists contains articles on many of the points above.

Software and code

Policy information about availability of computer code

Data collection	MS spectra were searched using the ddamsproteomics Nextflow pipeline (https://github.com/lehtiolab/ddamsproteomics, v2.11).
Data analysis	The R code used for the analyses is deposited at https://github.com/noorasissala/MS-Olink-comparison. The code for the PeptAffinity R Shiny app is deposited at https://github.com/isabelle-leo/PeptAffinity.

For manuscripts utilizing custom algorithms or software that are central to the research but not yet described in published literature, software must be made available to editors and reviewers. We strongly encourage code deposition in a community repository (e.g. GitHub). See the Nature Portfolio guidelines for submitting code & software for further information.

Data

Policy information about availability of data

All manuscripts must include a data availability statement. This statement should provide the following information, where applicable:

- Accession codes, unique identifiers, or web links for publicly available datasets
- A description of any restrictions on data availability
- For clinical datasets or third party data, please ensure that the statement adheres to our policy

The HiRIEF LC-MS/MS data have been deposited to the ProteomeXchange Consortium via the PRIDE partner repository with the dataset identifier PXD061144. The Olink Explore 3072 data have been deposited to the PRIDE repository with the dataset identifier PAD000006. Individual-level personal data are not publicly available

as they contain information that could compromise participants' privacy. Data used for the PeptAffinity R shiny app can be found in the code repository on GitHub. All other data supporting the findings of this study are available within the paper and its supplementary information files.

Research involving human participants, their data, or biological material

Policy information about studies with human participants or human data. See also policy information about sex, gender (identity/presentation), and sexual orientation and race, ethnicity and racism.

Reporting on sex and gender	Sex was included as a biological variable, determined from the Swedish personal identity number (assigned). No gender identity data were collected. The distribution of participants' sex is presented in Figure 1.
Reporting on race, ethnicity, or other socially relevant groupings	We did not gather information on race, ethnicity, or other socially relevant groupings.
Population characteristics	A summary of the population characteristics is provided in Figure 1 (age and sex) and Methods (diagnosis).
Recruitment	Participant recruitment has been described previously in Levitsky et al. Sci. Rep. 9, 16504 (2019). The studied cohort includes patients with lung symptomatology and lung-related diseases. Although the study cohort might have limited the generalizability of the findings with regards to protein detection, it is unlikely to have affected the agreement between proteomic platforms. However, including a study population that would be more heterogeneous with regards to disease distribution could further increase the variance in protein levels and potentially the agreement between platforms.
Ethics oversight	The study was approved by the Regional Ethical Review Board in Stockholm, Sweden (EPN: ref no 2014/1290–32).

Note that full information on the approval of the study protocol must also be provided in the manuscript.

Field-specific reporting

Please select the one below that is the best fit for your research. If you are not sure, read the appropriate sections before making your selection.

☒ Life sciences ☐ Behavioural & social sciences ☐ Ecological, evolutionary & environmental sciences

For a reference copy of the document with all sections, see nature.com/documents/nr-reporting-summary-flat.pdf

Life sciences study design

All studies must disclose on these points even when the disclosure is negative.

Sample size	The sample size was not calculated a priori. The correlations between MS and Olink were analysed on 88 samples, to fit within one Olink plate. The sample size was deemed sufficient for detecting a Pearson correlation coefficient of 0.294, given a staistical significance of α = 0.05 for a two-tailed Fisher z test and a power of 1-β=0.8. The Fisher transformation calculation for Pearson correlation coefficients was used as an approximation for the power for Spearman correlation coefficients, since the distribution under the null hypothesis behaves the same as Pearson correlation coefficients.
Data exclusions	No sample was excluded from analysis. Ten proteins in Olink data had values <LOD for all the samples and these proteins were excluded from further analyses; however, none were detected in the MS data, therefore the exclusion did not affect the cross-platform comparison. Data filtering in sensitivity analyses was performed at several instances, which is detailed in both the methods and results sections where applicable.
Replication	The findings regarding MS-Olink correlations were extensively replicated in 21 publicly available datasets generated with diverse affinity and MS proteomics methods. The findings regarding plasma proteome sex differences were also replicated in four publicly available datasets (two analyzed with Olink, one with Somascan, and one with MS proteomics).
Randomization	Samples were randomized when allocating to the Olink plate, and block randomized into TMT sets to ensure approximately equal numbers of lung cancer and non-cancer samples in each TMT set.
Blinding	Blinding was not applicable.

Reporting for specific materials, systems and methods

We require information from authors about some types of materials, experimental systems and methods used in many studies. Here, indicate whether each material, system or method listed is relevant to your study. If you are not sure if a list item applies to your research, read the appropriate section before selecting a response.

Materials & experimental systems

n/a	Involved in the study
☒	☐ Antibodies
☒	☐ Eukaryotic cell lines
☒	☐ Palaeontology and archaeology
☒	☐ Animals and other organisms
☒	☐ Clinical data
☒	☐ Dual use research of concern
☒	☐ Plants

Methods

n/a	Involved in the study
☒	☐ ChIP-seq
☒	☐ Flow cytometry
☒	☐ MRI-based neuroimaging

Plants

Seed stocks

Report on the source of all seed stocks or other plant material used. If applicable, state the seed stock centre and catalogue number. If plant specimens were collected from the field, describe the collection location, date and sampling procedures.

Novel plant genotypes

Describe the methods by which all novel plant genotypes were produced. This includes those generated by transgenic approaches, gene editing, chemical/radiation-based mutagenesis and hybridization. For transgenic lines, describe the transformation method, the number of independent lines analyzed and the generation upon which experiments were performed. For gene-edited lines, describe the editor used, the endogenous sequence targeted for editing, the targeting guide RNA sequence (if applicable) and how the editor was applied.

Authentication

Describe any authentication procedures for each seed stock used or novel genotype generated. Describe any experiments used to assess the effect of a mutation and, where applicable, how potential secondary effects (e.g. second site T-DNA insertions, mosiacism, off-target gene editing) were examined.
